# PED/PEA-15 Controls Fibroblast Motility and Wound Closure by ERK1/2-Dependent Mechanisms

**DOI:** 10.1002/jcp.22944

**Published:** 2011-07-21

**Authors:** Roberta Buonomo, Ferdinando Giacco, Angela Vasaturo, Sergio Caserta, Stefano Guido, Valentina Pagliara, Corrado Garbi, Gelsomina Mansueto, Angela Cassese, Giuseppe Perruolo, Francesco Oriente, Claudia Miele, Francesco Beguinot, Pietro Formisano

**Affiliations:** 1 Department of Cellular and Molecular Biology and Pathology, “Federico II” University of NaplesNaples, Italy; 2 Institute of Experimental Endocrinology and Oncology of CNRNaples, Italy; 3 Department of Chemical Engineering, “Federico II” University of NaplesNaples, Italy; 4 CEINGE Biotecnologie AvanzateNaples, Italy; 5 Department of Biomorphological and Functional Sciences, “Federico II” University of NaplesNaples, Italy

## Abstract

Cell migration is dependent on the control of signaling events that play significant roles in creating contractile force and in contributing to wound closure. We evaluated wound closure in fibroblasts from mice overexpressing (TgPED) or lacking ped/pea-15 (KO), a gene overexpressed in patients with type 2 diabetes. Cultured skin fibroblasts isolated from TgPED mice showed a significant reduction in the ability to recolonize wounded area during scratch assay, compared to control fibroblasts. This difference was observed both in the absence and in the presence of mytomicin C, an inhibitor of mitosis. In time-lapse experiments, TgPED fibroblasts displayed about twofold lower velocity and diffusion coefficient, as compared to controls. These changes were accompanied by reduced spreading and decreased formation of stress fibers and focal adhesion plaques. At the molecular level, TgPED fibroblasts displayed decreased RhoA activation and increased abundance of phosphorylated extracellular signal-regulated kinase 1/2 (ERK1/2). Inhibition of ERK1/2 activity by PD98059 restored RhoA activation, cytoskeleton organization and cell motility, and almost completely rescued wound closure of TgPED fibroblasts. Interestingly, skin fibroblasts isolated from KO mice displayed an increased wound closure ability. In vivo, healing of dorsal wounds was delayed in TgPED and accelerated in KO mice. Thus, PED/PEA-15 may affect fibroblast motility by a mechanism, at least in part, mediated by ERK1/2. J. Cell. Physiol. 227: 2106–2116, 2012. © 2011 Wiley Periodicals, Inc.

Cell migration is a dynamic process that requires coordinated cellular activities. It is inevitable for normal development and homeostasis and can also contribute to important phenomena including tissue repair (Olson and Nordheim, 2010). Cell migration can be subdivided into four phases: polarization of the cell in response to an external stimulus, formation of protrusion at the leading edge, adhesion to other cells or the extracellular matrix, and retraction of the trailing edge, which moves the cell forward (Ulrich and Heisenberg, 2009). Cell adhesion, migration, and contraction play significant roles in creating contractile force of wound margins and in contributing to wound closure. Thus, misregulation in one of these cell functions may have severe consequences and may impair wound-healing process (Sibbald and Woo, 2008). Altered wound healing is a significant cause of morbidity and mortality for a large portion of the adult population worldwide (Edmonds, 2004). One of the most common conditions associated with impaired wound healing is diabetes mellitus. About 15% of patients with diabetes present ulcers at lower extremities, quite difficult to heal (Trousdale et al., 2009). Multiple factors contribute to deficient healing in a subset of diabetic patients (Braiman-Wiksman et al., 2007; Trousdale et al., 2009). They include altered host response, diminished anti-bacterial defences, prolonged inflammation, altered protease activity, tendency for vascular abnormalities, generation of an inadequate number of cells to accomplish rapid and robust healing, decreased growth factor production, failure to form a sufficient amount of extracellular matrix, and alterations in apoptosis that may interfere with healing by decreasing the number of cells that participate in new tissue formation (Galkowska et al., 2006; Velander et al., 2008; Wall et al., 2008; Peppa et al., 2009; Schultz and Wysocki, 2009; Siqueira et al., 2010). In particular, fibroblasts play a pivotal role in tissue repair. They function both as synthesizer cells, depositing collagen-rich matrix, and as signaling cells, secreting growth factors important for cell–cell communication during the repair process (Falanga, 2005; Giacco et al., 2006). Any impediment to fibroblast functions prevents normal wound closure and results in chronic nonhealing wounds (Lerman et al., 2003). Noteworthy, alterations of fibroblast functions have been reported in individuals with type 2 diabetes (Lerman et al., 2003).

*PED/PEA-15* is a gene overexpressed in several tissues and cell types, including fibroblasts, of a large cohort of patients with type 2 diabetes (Condorelli et al., 1998; Condorelli et al., 2001; Valentino et al., 2006). PED/PEA-15 gene product is a ubiquitously expressed protein, which has been implicated in the control of cell survival and growth and glucose metabolism (Fiory et al., 2009). PED/PEA-15 lacks enzymatic function and mainly serves as a molecular adaptor. Indeed, it has been identified as an interactor for several signaling molecules including phospholipase D1 (Zhang et al., 2000), p90 ribosomal S6 protein kinase 2 (RSK2) (Vaidyanathan and Ramos, 2003), and extracellular signal regulated kinase 1/2 (ERK1/2) (Condorelli et al., 2002; Gaumont-Leclerc et al., 2004; Krueger et al., 2005; Gervais et al., 2006; Glading et al., 2007; Roth et al., 2007; Eckert et al., 2008). In particular, PED/PEA-15 binding to ERK1/2 prevents its nuclear translocation and determines cytosolic accumulation, thereby modifying its targeting to specific subsets of substrates (Formstecher et al., 2001).

We now show that the increase of PED/PEA-15 expression results in a reduced fibroblast motility, at least in part, by an ERK1/2-dependent mechanism. This may also contribute to defect of wound healing in TgPED mice.

## Materials and Methods

### Materials

Tribromoethanol (Avertin®) was from Sigma–Aldrich (St. Louis, Mo). Media, sera, and antibiotics for cell culture were purchased from Invitrogen Ltd. (Paisley, UK). Rabbit polyclonal ERK1/2 (clone C-16G, used at 1:2,000 dilution) and mouse monoclonal RhoA (clone 26C4, used at 1:2,000 dilution) antibodies were from Santa Cruz Biotechnology (Santa Cruz, CA), and antibodies toward the phosphorylated forms of ERK1/2 (used at 1:2,000 dilution) were from Cell Signal Technology (Beverly, MA). Rabbit polyclonal Paxillin antibodies (used at 1:100 dilution) were from Zymed Laboratories (S. San Francisco, CA). Rabbit polyclonal H3-histone and β subunit of the insulin-like growth factor-1 receptor antibodies were from Upstate (Millipore Corporation, Bedford, MA; clone 1-2 used at 1:1,000 dilution). Western blotting, ECL reagents were from Amersham (Arlington Heights, IL). Electrophoresis reagents were from BioRad (Richmond, VA). Rabbit polyclonal β-actin antibodies (clone I-19 used at 1:2,000 dilution), Bisindolylmaleimide (BDM), PD98059 and mytomicin C were from Sigma–Aldrich.

### Isolation and Culture of Fibroblasts, Western Blot, Cell Fractionation

TgPED, ped/pea-15 null (KO), and Wt control mice have been generated and characterized as described previously (Kitsberg et al., 1999; Vigliotta et al., 2004). Skin fibroblasts were obtained by punch biopsy from the animals. Cultures were established and grown as previously described (Atala and Lanza, 2001), and used between generation 3 and 10. Human skin fibroblasts were obtained by punch biopsies as previously described (Condorelli et al., 1998). For Western blotting, the cells were solubilized in lysis buffer (50 mM HEPES pH 7.5, 150 mM NaCl, 4 mM EDTA, 10 mM Na_4_PO_7_, 2 mM Na_3_VO_4_, 100 mM NaF, 10% glycerol, 1% Triton X-100, 1 mM phenylmethylsulfonyl fluoride, 100 µg of aprotinin/ml, 1 mM leupeptin) for 60 min at 4°C. Lysates were clarified at 5,000*g* for 15 min. Solubilized proteins were then separated by SDS–PAGE and transferred onto 0.45-µm-pore-size Immobilon-P membranes (Millipore Corporation). Upon incubation with the primary and secondary antibodies, immunoreactive bands were detected by ECL according to the manufacturer's instructions. For cell fractions, the cells were solubilized in ice-cold fractionation buffer (20 mM HEPES-NaOH, pH 7.4, 250 mM sucrose, 25 mM sodium fluoride, 1 mM sodium pyrophosphate, 0.1 mM sodium orthovanadate, 2 µM microcystin LR, 1 mM benzamidine) by passing them 10 times through a 22-gauge needle. Lysates were centrifuged at 800*g* for 5 min at 4°C. The nucleus pellet was solubilized in Buffer B (400 mM NaCl, 2 mM Na_3_VO_4_, 1 mM EGTA, 1 mM DTT) while supernatants were further centrifuged at 100,000*g* for 20 min at 4°C. The final supernatants represented the cytosolic fraction. The membrane pellet was solubilized in Buffer A containing 1% Triton X-100 and further centrifuged at 12,000*g* for 10 min at 4°C.

### Measurement of Rho Activity

Cellular Rho activity was measured by pull-down assay using the Rho binding domain of Rhotekin fused to GST (GST-RBD). For determining Rho activity, cells were extracted in Tris buffer (1% Triton X-100, 150 mM NaCl, 10 mM MgCl_2_, 10 mg/ml leupeptin, 10 mg/ml aprotinin, 1 mM PMSF). Rho activity (percentage of GTP-bound Rho) is determined as the amount of RBD-bound Rho versus total Rho in the lysate (Vial et al., 2003).

### Scratch Assay

Fibroblasts were seeded at a density of 8 × 10^5^ cells per well into the uncoated six-well microplates, wounded by manually scratching with a pipette tip, washed twice with phosphate-buffered saline (PBS) and incubated at 37°C, with or without mitomycin C (10 µg/ml; Sigma–Aldrich), PD98059 (30 µM; Sigma–Aldrich) or BDM (5 µM Sigma–Aldrich), as indicated in individual experiments. Images of wound gap were taken at 0 and 24 h by a Canon Powershot digital camera coupled to the microscope and percentage of closure was calculated with NIH IMAGE J. These experiments were repeated at least three times.

### Time-Lapse Microscopy

Time-lapse microscopy (TLM) experiments were performed using an inverted microscope (Zeiss Axiovert 200, Carl Zeiss, Oberkochen Germany), with a long working distance 10× objective in phase contrast, as described elsewhere (Fico et al., 2008). Briefly, the microscope is equipped with motorized stage and focus (Prior, Cambridge, UK) for automated sample positioning, and an incubator consisting of a plastic enclosure kept at 37 ± 0.1°C by a warm air flux, tuned by a PID controller. Furthermore, air premixed with 5% CO_2_ is blown trough a bubbling column for humidity saturation, and fed to a small chamber surrounding the sample to prevent water evaporation and pH changes in the cell culture medium. The images are captured by a cooled monochromatic CCD video camera (Hamamatsu, Japan). All the equipment are driven by a Labview macro that iteratively acquires images of selected regions every 10 min (Δt), for overall 24 h. The scratch assay (described above) was carried out in the TLM experiments and cell motion was analyzed offline as described in the following.

### Cell Tracking

Image analysis of the scratch assay was performed using a semi-automated Cell Tracking software. For each time step, about 40 cells on the wound edges were individually followed by manual overlaying each cell contour, and the coordinates of the contour points and of the cell center of mass were stored on hard disk. To assist the operator in cell identification, the color-coded contour of each cell at the previous time step is also shown in the image overlay. The trajectory of each cell was reconstructed for the whole experiment starting from the center of mass coordinates. Furthermore, average motility parameters of the cell population, such as velocity, were calculated as a function of time. The analysis of cell motility was based on the persistent random walk theory (Matthes and Gruler, 1988; Dickinson et al., 1994), where it is assumed that cell motion is characterized by a diffusion coefficient (also referred to as the random motility coefficient) D (µm^2^/min) and a persistence time P (min), that is, the characteristic time in which cell movement persists in the same direction. The value of D is a quantitative measurement of cell migration, and is related both to the average speed of the cells and to the persistence time. According to the theory (Uhlenbeck and Ornstein, 1930; Dickinson and Tranquillo, 1993), the mean square displacements are given by the equation


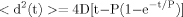
(1)

where <d^2^(t)> (µm^2^) is the mean square displacement of the tracked cell sample at time t. The trend predicted by Equation (1) is linear at t ≫ P (i.e., <d^2^(t)> ≍ 4Dt), with a slope proportional to the diffusion coefficient. The mean square displacements are calculated from the following relation


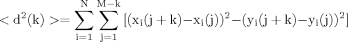
(2)

where k is the current time expressed in units of the time interval Δt between two consecutive image acquisitions, the double summation is on the index i representing cell number (up to the total cell number N) and on index j representing the number of intervals (the total number being M-k), x and y are the center of mass coordinates obtained by cell tracking. The mean square displacements are calculated from nonoverlapping intervals (Dickinson and Tranquillo, 1993; Dickinson et al., 1994). Equation (1) was fit to the experimental data of mean square displacements (calculated from Eq. 2) as a function of time with D and P as the only adjustable parameters.

As a further parameter of the cell movement we calculated the fraction of mobile cells, defined as the cells showing a total displacement higher than 200 µm, that is, at least equal to half of the wound width.

### Cell Adhesion and Cytoplasmic Spreading Assays

For cell adhesion assays, tissue culture wells were coated with 10 ng/ml of fibronectin (Sigma–Aldrich) at 37°C overnight. After saturation with 1% BSA, equal numbers of cells were seeded. Cells in DMEM were allowed to adhere for 1 h at 37°C, fixed with ethanol and stained with 0.5% crystal violet for 15 min. Images of adherent cells were captured and quantified by measuring the optical density at 540 nm with a microtiter plate reader. For cell spreading, fibroblasts were seeded at a density of 1 × 10^4^ cells per well into the 12-well microplates coated with fibronectin (1 µg/well) or heat-denatured BSA, as a negative control. After 3 h, cells were gently washed with PBS and fixed in 4% formaldehyde for 15 min. After staining with 0.1% crystal violet, the cells were examined by microscopy. Five randomly chosen visual fields, representing approximately 15% of the dish surface area, were photographed and the number of cells was counted (at least 1,000 cells). The cells were scored as either nonspread or spread (cells that had become flattened with their total diameter more than twice the diameter of the nucleus) (Enserink et al., 2004).

### Confocal Microscopy

Sub-confluent cells on glass coverslips were fixed for 20 min with 4% paraformaldehyde Sigma–Aldrich (St. Louis, Mo) in PBS containing 0.9 mM calcium and 0.5 mM magnesium (PBS-CM) at room temperature, washed twice in 50 mM NH_4_Cl in PBS-CM and twice in PBS-CM. Cells were permeabilized for 5 min in 0.5% Triton-X 100 (Bio-Rad) in PBS-CM, washed twice, for 10 min, in 0.2% gelatin (Sigma) in PBS-CM and then incubated for 1 h with anti-paxillin antibodies diluted in 0.5% BSA (Sigma) in PBS. After three washes with 0.2% gelatine, cells were incubated for 20 min with fluorescein-tagged goat anti-mouse secondary antibody (Jackson ImmunoResearch, West Grove, PA), diluted 1:50 in 0.5% BSA in PBS. For actin staining, rhodamin-conjugated phalloidin was used at the dilution of 1:200. After final washes with PBS, the coverslips were mounted on a microscope slide using a 50% solution of glycerol in PBS and examined with a Zeiss LSM 510 version 2.8 SP1 Confocal System. The experiments were done with or without treatment with 30 µM PD98059.

### In Vivo Wound Healing and Histological Analysis

We used 16 eight month-old, sex-, and age-matched, TgPED and Wt littermates and eight KO mice for each time point of the study. The animals were anesthetized with a single intraperitoneal injection of tribromoethanol (Avertin® 250 mg/kg body weight). The hair on the back of each mouse was cut and two full-thickness wounds (width about 4 mm, length about 2 cm) were made with scalpel (Braiman-Wiksman et al., 2007). Wounds from all animals were harvested at 3, 4, and 6 days after injury and used for histological analysis. Samples were fixed in 10% neutral buffered formalin and subsequently processed, blocked, and sectioned perpendicularly to the wound surface in 5 µm consecutive sections. Healing of skin wounds was evaluated on hematoxylin–eosin (H&E) slides under digital microscope (LEICA DMD108) at 5× magnification, by measuring the distance between migration tongues using the measurement tool included in the digital microscope software. Four sections of wound specimen were analyzed for each animal. Granulation tissue-staining of proliferating cells with antibodies anti-proliferating cell nuclear antigen (PCNA), collagen fibers with Masson–Trichrom, and H&E staining were used to assess the granulation-tissue formation. Granulation tissue was considered fully formed (100%) when the following parameters existed at wound gap: (1) a continuous layer of granulation tissue formed across the entire wound gap; (2) the layer of granulation tissue filled the entire wound depth. Wounds exhibiting sporadic formation patterns were considered negative for granulation tissue formation (Braiman-Wiksman et al., 2007). The inflammatory infiltrate was estimated by immunohistochemical-based quantification of neutrophils, macrophages, and T lymphocytes with anti-neutrophil antibody, anti-CD68 antibody, and anti-CD3 antibody, respectively (Hattori et al., 2009). PicroSirius Red/Fast Green was used for differential staining of collagen during matrix production phase. FGF-2 (1:500 dilution; sc-79, Santa Cruz Biotechnology) was used for immunohistochemical-based quantification of fibroblast content. All stained samples were examined under digital and light microscope by at least two trained pathologists in blind. The content of fibroblasts, infiltrated cells and collagen fibers was scored using a semi-quantitative three-point scale of range values. For fibroblasts and cellular infiltration, we attributed score 0 for no increase, score 1, 2, or 3 for little, moderate, or high increase of cell content compared to adjacent tissue, respectively. For the extracellular matrix production we attributed score 0 for absence of collagen production, score 1 and 2 for 10–40% and 40–80% collagen fibers content compared to adjacent normal tissue, respectively, and finally score 3 for wound matrix indistinguishable from adjacent normal tissue. All experiments were performed according to the guidelines and approved by the local Ethic Committee.

## RESULTS

### PED/PEA-15 Effect on Wound Closure

**Fig. 1:**
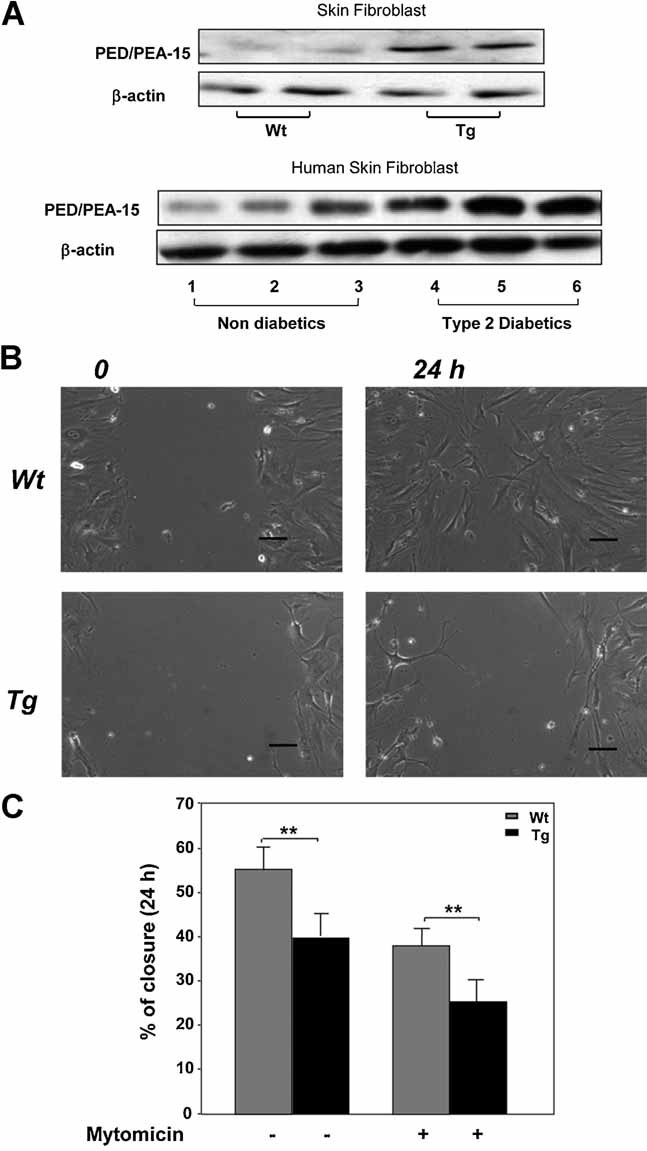
Wound healing in cultured fibroblasts from TgPED mice. A: Protein lysates (50 µg) of cultured fibroblasts from Wt and TgPED mice (top panel) and of skin fibroblasts obtained from three nondiabetic individuals (**lanes 1**–**3** in the bottom panel) and three individuals affected by type 2 diabetes (**lanes 4**–**6** in the bottom panel) were subjected to SDS–PAGE and immunoblotted with anti-PED/PEA-15 and β-actin antibodies. Blots were revealed by ECL and autoradiography. The blot shown is representative of four independent experiments. B: Confluent monolayers of fibroblasts from Wt and TgPED mice were subjected to scratch assays, as described in “Materials and methods” Section. Phase contrast microscopy images of cultured fibroblasts from TgPED mice and their Wt controls in the scratch assay at the beginning 0 and after a 24 h time-lapse experiment 24 h are shown. For all the panels, scale bar is 100 µm. C: Scratch assays were performed in the absence or in the presence of 10 µg/ml Mitomycin C, as indicated. Healing was calculated as described in “Materials and methods” Section. Bars represent the mean ± SD of triplicate determination in four independent experiments. Asterisks denote statistically significant differences (**P < 0.01).

Wound closure was studied in skin fibroblasts isolated from mice overexpressing *PED/PEA-15* gene (TgPED) and in their wild type (Wt) littermates. As expected, PED/PEA-15 protein abundance was about 10-fold higher in the TgPED fibroblasts (Fig. 1A). In TgPED mice, the increase of PED/PEA-15 levels was comparable to that found in cultured fibroblasts derived from skin biopsies of patients affected by type 2 diabetes, compared to unaffected individuals (Fig. 1A). Confluent monolayers of mouse fibroblasts were scratched and images were taken at 0 and 24 h after wounding (Fig. 1B). The wound closure was significantly decreased in TgPED fibroblasts compared to controls (Fig. 1C).

To determine whether the effect of PED/PEA-15 was due to alteration of cell proliferation, scratch assays were also performed in the presence of 10 µg/ml mitomycin C, an irreversible inhibitor of mitosis. Treatment with mitomycin C decreased the wound closure rate in fibroblasts of both genotypes. However, the extent of closure of TgPED fibroblasts was still significantly reduced compared to controls (Fig. 1C). Moreover, no difference between Wt and TgPED fibroblasts was detected in thymidine incorporation experiments (data not shown), suggesting that PED/PEA-15 effect on wound closure was not due to changes in cell proliferation.

### Direct Evaluation of Fibroblast Motility by Time-Lapse Microscopy (TLM)

**Fig. 2:**
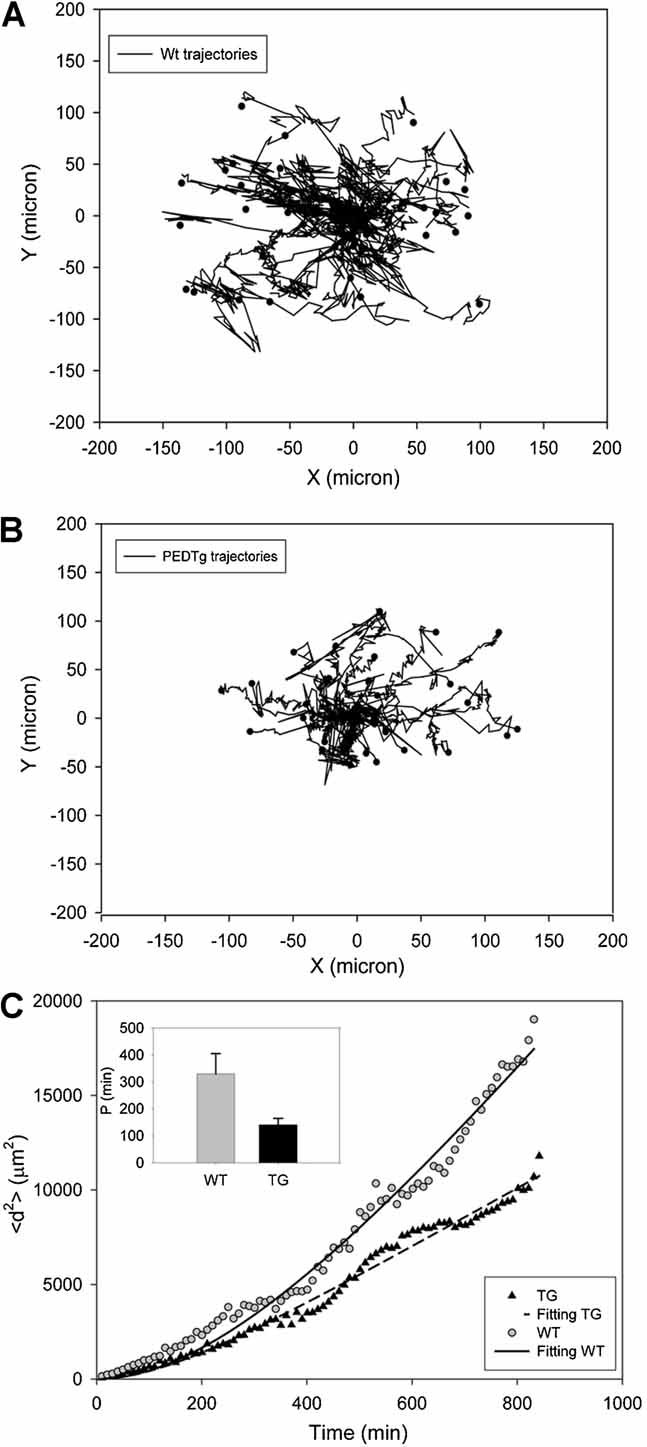
Fibroblast trajectory analysis and mean square displacements. A direct analysis of cell trajectories is used to characterize the motion of Wt (A) and TgPED fibroblasts (B). The trajectory of each cell is represented by the sequence of the cell center of mass positions translated to start from the same origin. To quantitatively assess cell movement, the mean square displacements (C) are calculated for a population of 40 cells tracked for 16 h using a semiautomated Cell Tracking software as described in “Materials and methods” Section. The inset in (C) reports the different persistence time calculated for the WT and TgPED.

Fibroblast motility was then assessed by quantitative analysis of images acquired in TLM experiments (see “Materials and methods” Section) following the scratch and images were recorded with a time interval of 10 min for 24 h. Videos of 24 h TLM experiments are available as supplementary material (TgPED and Wt). Wound closure was visually almost complete in 24 h for Wt cells, whereas TgPED fibroblasts were lagging behind. In agreement with the striking difference in wound closure rate, TgPED fibroblasts exhibited a significant lower average velocity (Table 1). Moreover, cell trajectories were reconstructed by the cell tracking image analysis (see “Materials and methods” Section). In both Wt (Fig. 2A) and TgPED (Fig. 2B) fibroblasts, the trajectories showed a random orientation being uniformly distributed in space (i.e., no preferential direction of motion can be distinguished). However, more extended cell trajectories were detected in Wt compared to TgPED fibroblasts (Fig. 2A,B), in agreement with the higher fraction of cells (84% and 42% for Wt and TgPED, respectively) that showed a displacement at least equal to half the wound size (Table 1). The mean square displacements <d^2^(t)> of the two populations were calculated from the cell centers of mass at each time (see Eq. 2 in “Materials and methods” Section). The <d^2^(t)> values, which are representative of cell migration by random diffusion, were about twofold higher in Wt fibroblasts (Fig. 2C). Accordingly, the values of D (diffusion coefficient) and P (persistence time, Fig. 2C, inset) were about twofold higher in Wt compared to TgPED fibroblasts (Table 1).

**TABLE 1:** Assessment of motility parameters in fibroblasts from Wt and TgPED mice

Genotype	Velocity (µm/min)	Fraction of mobile cells	D (µm^2^/min)	*P* (min)
Wt	0.9 ± 0.1	84 ± 6%	8 ± 3	330 ± 75
TgPED	0.5 ± 0.1^*^	42 ± 5%^*^	4 ± 1^*^	140 ± 25^*^

The average cell velocity, the fraction of mobile cells (i.e., cells moving at least half the wound width) the value of D (diffusion coefficient) and P (persistence time) were calculated, as described in “Materials and methods” Section, from the mean square displacements of Fig. 2C for both Wt and TgPED fibroblasts. For each sample the movement of about 40 cells was tracked for each time step. Data are statistically significant (^*^P < 0.01).

### PED/PEA-15 Effect on Cell Adhesion, Spreading, and Cytoskeleton Organization

**Fig. 3:**
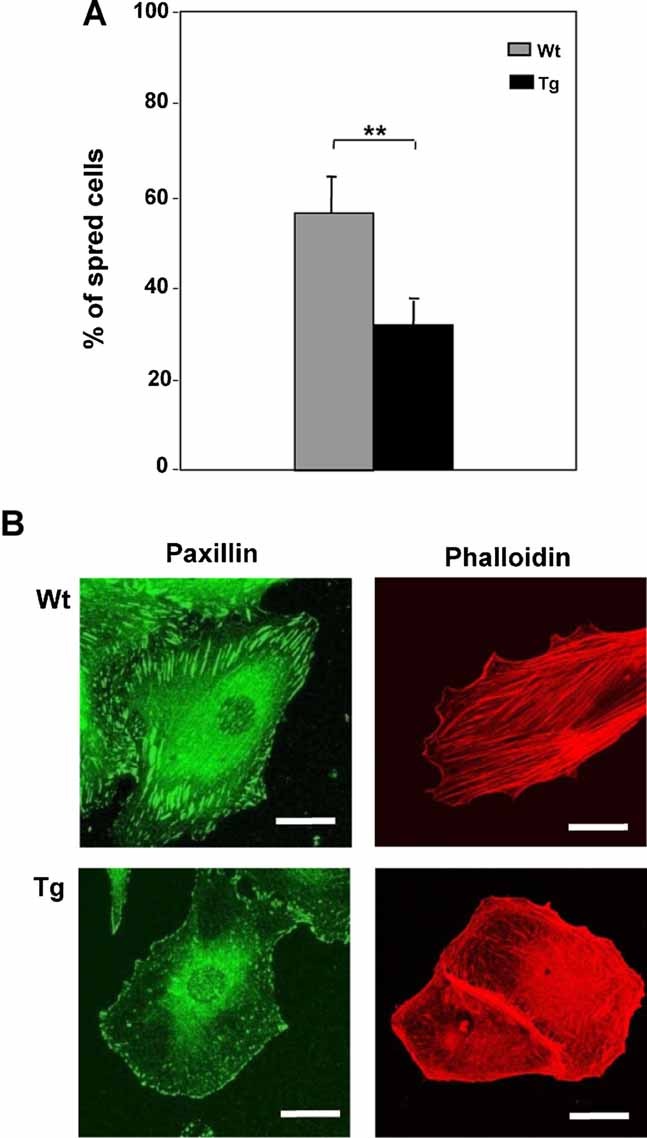
Cytoplasmic spreading and cytoskeleton organization in Wt and TgPED fibroblasts. A: Fibroblasts from Wt and TgPED mice were subjected to spreading assays. After 3 h plating on fibronectin, cytoplasmic spreading was quantified by light microscope after staining with crystal violet as described in “Materials and methods” Section. Bars represent the mean ± SD of three independent determinations in triplicate. Asterisks denote statistically significant differences (**P < 0.01). B: Immunofluorescence analysis was performed as described in “Materials and methods” Section. Focal adhesion plaques formation were investigated by immunostaining with specific anti-paxillin antibody. Stress fibers organization were detected by rhodaminate–phalloidin staining. The experiment was repeated four times with similar results. Representative images are shown. The scale bar is 15 µm.

Fibroblast motility is dependent on cell ability to adhere to substrates and to transfer into the cytoplasm the signal that induces the cytoskeleton reorganization (Ridley et al., 2003; Arnaout et al., 2007). TgPED fibroblasts showed no significant difference in adhesion compared to the controls (data not shown). Moreover, after 3 h plating on fibronectin, about 60% of Wt cells exhibited a spread cytoplasm. By contrast, the number of spread cells was about 30% for TgPED (Fig. 3A). To analyze actin cytoskeleton and organization of focal adhesion plaques, Wt and TgPED fibroblasts were stained with rhodamin-conjugated phalloidin or with specific anti-paxillin antibodies, respectively. Clearly, the results showed that at least 70% of TgPED fibroblasts displayed a marked decrease of stress fibers and focal adhesion plaques compared to controls (Fig. 3B).

### PED/PEA-15 Effect on RhoA Activity and Subcellular Distribution

**Fig. 4:**
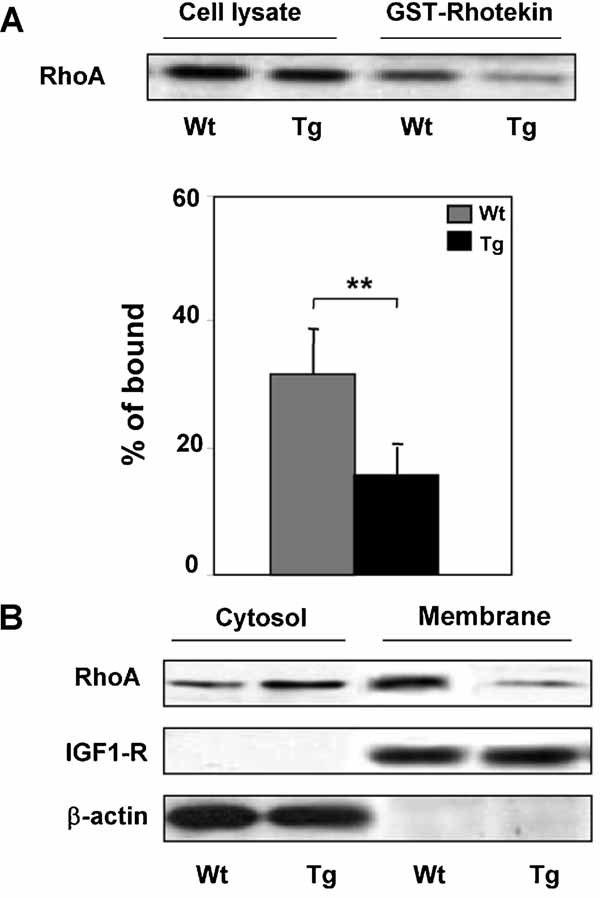
RhoA activity and localization in fibroblasts from Wt and TgPed fibroblasts. A: Cell lysates were obtained from Wt and TgPED fibroblasts as described in “Materials and methods” Section. Pull-down assays with GST-Rhotekin were performed and analyzed by Western blot. Blots were revealed by ECL and autoradiography. Densitometry for pulled-down RhoA-GTP was normalized to the amount of total RhoA. The results are presented as percentage of bound respect to total level of protein. Data are mean of four independent experiments. Asterisks denote statistically significant differences (**P < 0.01). B: Cytosolic and membrane fractions of Wt and TgPED fibroblasts were obtained as described in “Materials and methods” Section, subjected to SDS–PAGE and immunoblotted with anti-RhoA, β-actin and IGF-1 receptor β-subunit antibodies, as indicated. Blots were revealed by ECL and autoradiography. The blots shown are representative of four independent experiments.

RhoA-GTPase is a protein that plays a major role in the organization of stress fibers and in the formation of focal adhesion plaques in the cytoplasm (Huveneers and Danen, 2009). When released from Rho GDP dissociation inhibitors (GDIs), Rho-GTPases are targeted to the plasma membrane, where its activation cycle is regulated by guanine-exchange factors (GEFs) that promote GTP loading and activation of Rho-GTPases (Raftopoulou and Hall, 2004). RhoA activity was assessed in pull down assay by measurement of the levels of active GTP-bound protein. In TgPED fibroblasts the levels of active RhoA were decreased with no change in RhoA expression compared to Wt cells (Fig. 4A). Moreover, RhoA membrane localization was markedly decreased in TgPED fibroblasts compared to control cells (Fig. 4B). Consistently, RhoA cytosolic detection was much stronger in TgPED than in Wt fibroblasts, suggesting an aberrant localization of the protein.

### PED/PEA-15 Effect on ERK 1/2 Activation and Function

**Fig. 5:**
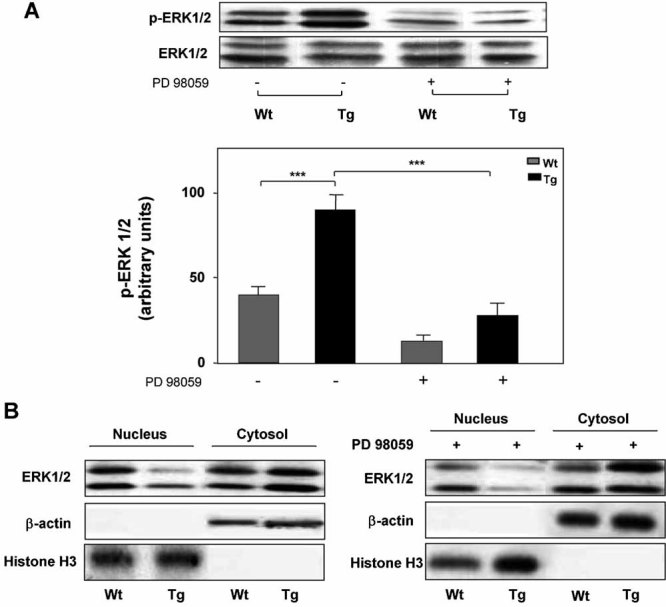
Evaluation of ERK 1/2 activation by PED/PEA-15 in Wt and TgPED fibroblasts. A: Lysates of Wt and TgPED fibroblasts were obtained before and after treatment with PD98059 as described in “Materials and methods” Section, subjected to SDS–PAGE and immunoblotted with anti-phospho-ERK1/2 and ERK1/2 antibodies. Blots were revealed by ECL and autoradiography. The blots shown are representative of four independent experiments. Autoradiographs of the experiments were subjected to densitometric analysis. B: Cytosolic and nuclear fractions of Wt and TgPED fibroblasts were obtained as described in “Materials and methods” Section, subjected to SDS–PAGE and immunoblotted with anti-ERK1/2, β-actin, and Histone H3 antibodies. Blots were revealed by ECL and autoradiography. The experiment was done in the absence or in the presence of 30 µM PD98059, as indicated. The blots shown are representative of four independent experiments.

**Fig. 6:**
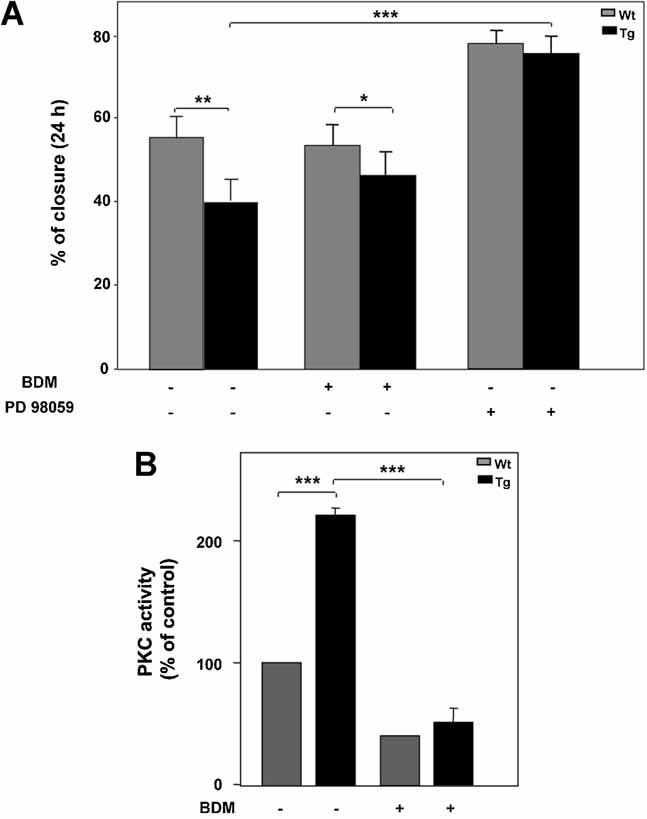
Effect of PKC and ERK inhibitors on in vitro wound healing. A: Confluent monolayers of fibroblasts from Wt and TgPED mice were subjected to scratch assays, as described in “Materials and methods” Section, and in the legend of Figure 1. The scratch assays were performed in culture medium alone or added with 5 µM BDM or 30 µM PD98059, as indicated. Healing was calculated as described in “Materials and methods” Section. Bars represent the mean ± SD of triplicate determination in four independent experiments. Asterisks denote statistically significant differences (*P < 0.05; **P < 0.01; ***P < 0.001). B: PKC activity was assayed as described in “Materials and methods” Section. PKC activity is expressed as percentage over control activity. Bars represent the mean ± SD of data from four independent experiments. Asterisks denote statistically significant differences (***P < 0.001)

**Fig. 7:**
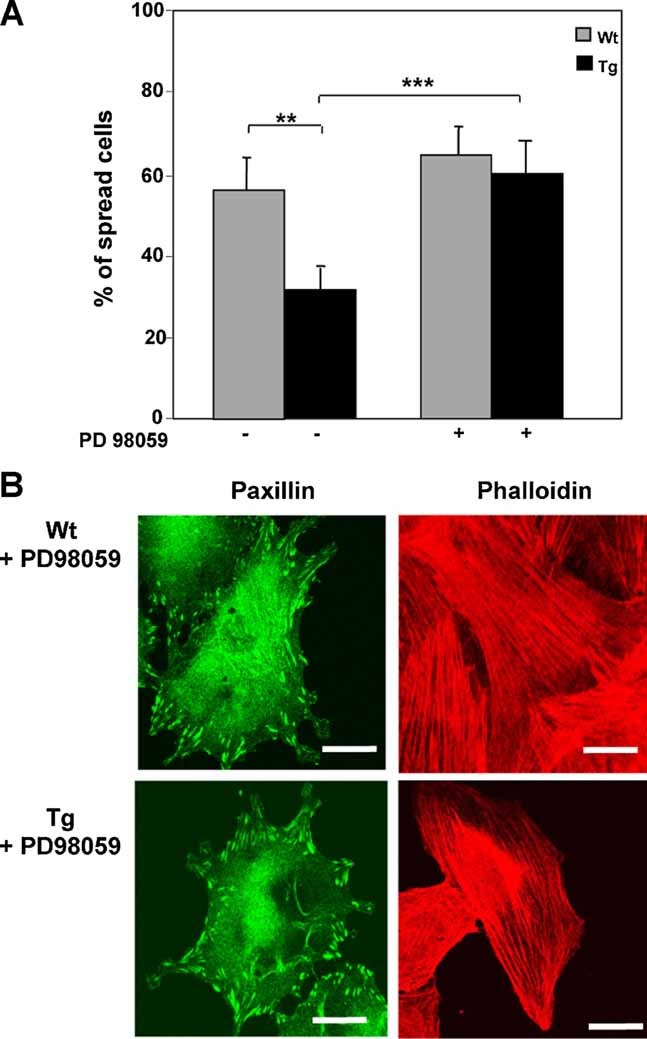
Effect of PD98059 on spreading and cytoskeleton organization and RhoA localization. A: Cytoplasmic spreading was quantified in TgPED fibroblasts pre-incubated in the absence or in the presence of 30 µM PD98059, as indicated, as described in “Materials and methods” Section and in the legend to Figure 3. Bars represent the mean ± SD of three independent determinations in triplicate. Asterisks denote statistically significant differences (**P < 0.01; ***P < 0.001). B: Immunoflorescence analysis was performed in Wt and TgPED fibroblasts incubated with 30 µM PD98059, as described in the legend to Figure 3. Focal adhesion plaques formation was investigated by immunostaining with specific anti-paxillin antibody. Stress fibers were detected by rhodaminate–phalloidin staining. The experiment was repeated four times with similar results. Representative images are shown. The scale bar is 15 µm.

**Fig. 8:**
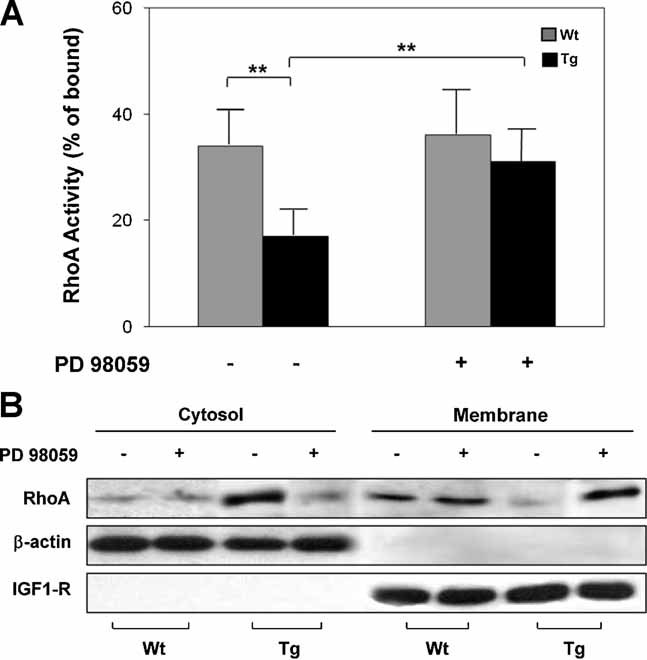
Effect of PD98059 on RhoA activity and localization. A: Cell lysates were obtained from Wt and TgPED fibroblasts as described in “Materials and methods” Section. Pull-down assays with GST-Rhotekin were performed and analyzed by Western blot as described in Figure 4. Densitometry for pulled-down RhoA-GTP was normalized to the amount of total RhoA. The results are presented as percentage of bound respect to total level of protein. Data are mean of four independent experiments. Asterisks denote statistically significant differences (**P < 0.01). B: Cytosolic and membrane fractions of Wt and TgPED fibroblasts cultured in the absence or in the presence of 30 µM PD98059, as indicated, were obtained as described in “Materials and methods” Section, subjected to SDS–PAGE and immunoblotted with anti-RhoA, β-actin, and IGF-1 receptor β-subunit antibodies. Blots were revealed by ECL and autoradiography. The blots shown are representative of four independent experiments.

It has been previously demonstrated that PED/PEA-15 increases the activation of ERK1/2 in CHO cells (Ramos et al., 2000). We have therefore tested ERK1/2 phosphorylation in Wt and TgPED fibroblasts. As expected, ERK1/2 activation was increased in TgPED fibroblasts compared with Wt fibroblasts (Fig. 5A). Moreover PED/PEA-15 overexpression increased ERK1/2 cytosolic localization in TgPED fibroblasts compared to the control cells (Fig. 5B). To assess whether ERK1/2 hyper-activation could be responsible for the alterations in wound closure, spreading, RhoA activation, and organization of cytoskeleton structures, fibroblasts from both Wt and TgPED mice were treated with 30 µM PD98059 (an inhibitor of the MEK-ERK1/2 pathway). At this concentration PD98059 did not affect proliferation of Wt and TgPED fibroblasts (data not shown), as well as ERK1/2 localization (Fig. 5B). Nevertheless it was able to reduce ERK 1/2 phosphorylation in both cellular types (Fig. 5A). Treatment with PD98059 increased wound closure in Wt fibroblasts and almost completely rescued wound closure in TgPED fibroblasts (Fig. 6A). At variance, in both Wt and TgPED fibroblasts, almost no effect was elicited by treatment with 5 µM bisindolylmaleimide (BDM), which inhibited PKC activity in both cell types (Fig. 6B). Moreover, treatment with PD98059 completely reverted the effect of PED/PEA-15 on spreading (Fig. 7A) and determined a substantial rescue of stress fibers and focal adhesion plaques in about 90% of TgPED fibroblasts, compared to the controls (Fig. 7B). Similarly, PD98059 treatment restored RhoA activity (Fig. 8A) and plasma membrane content in TgPED fibroblasts to the levels observed for Wt fibroblasts (Fig. 8B). At variance BDM treatment had no significant effect on cell spreading, on stress fibers and focal adhesion plaques and on RhoA activity and localization (data not shown).

### PED/PEA-15 Depletion Increased Fibroblast Spreading and Wound Closure

**Fig. 9:**
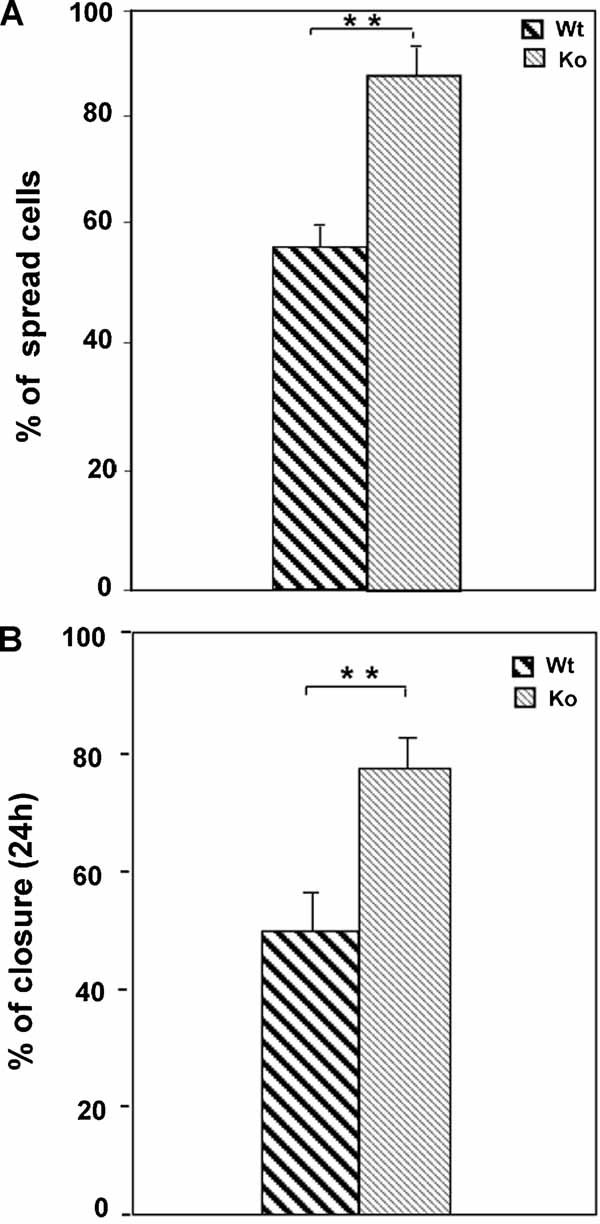
Cytoplasmic spreading and in vitro wound healing in *ped/pea-15* null fibroblasts. A: Fibroblasts from Wt and *ped/pea-15* null mice were subjected to spreading assays. After 3 h, cytoplasmic spreading on fibronectin was quantified by light microscope after staining with crystal violet as described in “Materials and methods” Section. Bars represent the mean ± SD of three independent determinations in triplicate. Asterisks denote statistically significant differences (**P < 0.01). B: Confluent monolayers of fibroblasts from Wt and *ped/pea-15* null mice were subjected to scratch assays, as described in “Materials and methods” Section. Healing was calculated as described in “Materials and methods” Section. Bars represent the mean ± SD of triplicate determination in four independent experiments. Asterisks denote statistically significant differences (**P < 0.01).

To further address the role of PED/PEA-15 in the regulation of cellular motility, we used fibroblasts from *ped/pea-15* null mice (KO). These animals have been previously characterized and reported (Miele et al., 2007) and feature no PED/PEA-15 expression in skin fibroblasts (data not shown). Importantly, 3 h after plating on fibronectin, cytoplasmic spreading was increased by about 30% compared to control cells (Fig. 9A). Likewise, the ability to recolonize the wounded area was also increased in *ped/pea-15* null fibroblasts (Fig. 9B; see also video in supplementary material). Furthermore, wound closure was almost complete after 16 h in *ped/pea-15* null fibroblasts, while it occurred at least after 24 h in control mice.

### PED/PEA-15 Effect on Wound Healing In Vivo

In order to assess whether PED/PEA-15 may also affect fibroblast motility in vivo, we examined wound healing in TgPED, in KO and in Wt mice. Dorsal skin was wounded as described in “Materials and methods” Section. After 4 days since dorsal incision, the distance between the wound edges was about threefold higher in TgPED mice and twofold lower in KO, compared to their respective wild-type littermates. TgPED mice also presented significantly reduced granulation tissue formation as compared to Wt (Table 2). Moreover, the content of fibroblasts in the healing area was significantly reduced in TgPED and increased in KO mice (Table 2). Decreased production of collagen fibers and increased detection of infiltrated inflammatory cells were also observed in TgPED mice.

**TABLE 2:** Assessment of wound healing in TgPED and KO mice 4 days post-wounding

Genotype	Epidermal closure (mm)	Granulation tissue thickness (%)	Collagen fibres production	Fibroblasts content	Infliltrated cells content
Wt	0.6 ± 0.6	70	1.4 ± 0.7	1.6 ± 1	1 ± 0.9
TgPED	1.7 ± 1.4 (*P* < 0.001)	40 (*P* < 0.001)	0.6 ± 0.9 (*P* < 0.001)	0.9 ± 1 (*P* < 0.01)	1.5 ± 1.3 (*P* < 0.01)
KO	0.4 ± 0.1 (*P* < 0.05)	ND	ND	1.9 ± 0.3 (*P* < 0.05)	ND

Skin specimens harvested 4 days after dorsal incisions from Wt (n = 16), TgPED (n = 16), and KO (n = 8) mice were examined and evaluated as described in “Materials and methods” Section (ND, not determined).

## DISCUSSION

The major driving force of cell migration is the extension of a leading edge protrusion, the establishment of new adhesion sites at the front, the cell body contraction, and the detachment of adhesions at the cell rear (Ridley et al., 2003). All these steps involve assembly, disassembly, or reorganization of the actin cytoskeleton, and each must be coordinated both in space and time to generate productive, net forward movement (Ridley et al., 2003). Cell migration is an essential process not only during development, but also in wound repair. Nevertheless, the molecular mechanisms involved in wound closure alterations in several pathological conditions, including diabetes (Terranova, 1991; Baum and Arpey, 2005), are not well understood.

PED/PEA-15 overexpression represents a common feature in skeletal muscle, adipose tissue, and fibroblasts from individuals with type 2 diabetes (Condorelli et al., 2001; Valentino et al., 2006). Moreover transgenic mice overexpressing *PED/PEA-15* (TgPED) to levels comparable to those occurring in type 2 diabetic patients, display altered glucose tolerance and impaired insulin secretion. Here, we show that overexpression of *PED/PEA-15* affects wound closure in cultured primary fibroblasts isolated from TgPED mice. TgPED fibroblasts displayed reduced average velocity compared to Wt and their trajectories were much shorter than those of Wt fibroblasts. Also, the reduced persistence time in the wounded area is coherent with the reduced motility displayed by TgPED fibroblasts. Conversely, fibroblasts from KO mice exhibited an accelerated wound closure. Cell interaction with the ECM components is crucial for adhesion and migration and is mediated by various receptors, including integrins (Arnaout et al., 2007). These proteins provide a transmembrane link between the ECM and the cytoskeleton, activating intracellular signaling processes (Huveneers and Danen, 2009). By analyzing cell adhesion we found no difference between TgPED and Wt (data not shown), while cytoplasmic spreading was significantly reduced in TgPED fibroblasts. Reduced spreading was paralleled by a decreased cellular content of focal adhesion plaques (FAPs) and actin stress fibers. Thus, recolonization of wounded area, spreading, and cytoskeleton organization are modulated by PED/PEA-15.

Rho GTPase family of proteins, including Ras-related C3 botulinum toxin substrate 1 (Rac1), cell division cycle 42, (Cdc42) and RhoA (Machacek et al., 2009), plays a pivotal role in regulating the biochemical pathways relevant to cell migration. In particular RhoA is a small GTPase switching between an inactive GDP-bound form, localized in the cytoplasm, and an active GTP-bound form, localized in the plasma membrane, where it coordinates and controls the formation of focal adhesion plaques and actin stress fibres (DeMali et al., 2003; Raftopoulou and Hall, 2004; Mitra et al., 2005). Initial cell adhesion and spreading occur in parallel with the activation of Rac1 and Cdc42, and determine enhanced actin-mediated protrusion of lamellipodia and filopodia. At a later phase, the activities of Rac1 and Cdc42 decrease, whereas the activity of RhoA gradually increases, determining the polymerization of actin stress fibers and the formation of focal adhesion plaques which allow cell migration (Raftopoulou and Hall, 2004). We have previously shown that PED/PEA-15 inhibits Cdc42 activation (Condorelli et al., 2002). This effect may, at least in part, explain the reduction of spreading in TgPED fibroblasts. Interestingly, RhoA membrane content was also reduced in TgPED compared to Wt fibroblasts, suggesting the involvement of PED/PEA-15 in the later phase of cell migration, as well.

Reduced RhoA activation determines an impairment of cytoskeletal structure formation, leading to an overall reduced cellular movement (Narumiya et al., 2009). In TgPED fibroblast, this is paralleled by a reduced content of stress fibers and FAPs. Although both stress fibers and FAPs may be also required for cell anchorage, which would have a negative correlation with cellular motility, the impaired dynamic formation of such cytoskeletal structures may devoid the cell of driving force (Small et al., 1999), needed for cell movement and explain, at least in part, the reduced motility displayed by TgPED fibroblasts.

Different signals from the cell exterior can modulate RhoA function. For example, integrin-mediated adhesion induces the activation of extracellular signal regulated kinase/mitogen-activated protein kinase (ERK/MAPK) signaling, which, in turn, regulates the function of Rho family members involved in the cytoskeleton dynamics. Moreover, activated ERK1/2 localizes to focal adhesion where it phosphorylates several substrates promoting the dynamic turnover of the structure necessary for the movement (Pullikuth and Catling, 2007).

Here we show that ERK1/2 cytoplasmic activity is increased in TgPED compared to Wt fibroblasts, as it was previously shown in other cell types (Ramos et al., 2000).

**Fig. 10:**
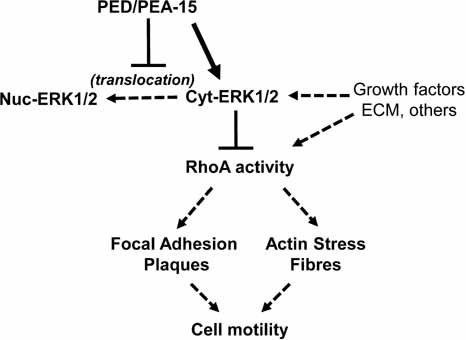
Schematic representation of PED/PEA-15 effect on cytoskeletal organization and cell motility. Many growth factors, extracellular matrix proteins, and other extracellular signals induce ERK1/2 activation and translocation from cytosol (Cyt-ERK1/2) to nucleus (Nuc-ERK1/2), as well as RhoA activity and cytoskeletal rearrangements allowing cellular motility. When PED/PEA-15 is hyper-expressed, it binds ERK1/2 and prevents nuclear translocation, thereby causing accumulation into the cytosol. This is paralleled by a decrease of RhoA activity, which most likely affects the formation of FAPs and stress fibers. In cells overexpressing PED/PEA-15, biochemical and morphological changes are also accompanied by an overall reduced cellular motility.

Inhibition of ERK1/2 activity with PD98059 rescued RhoA GTP loading and membrane recruitment, formation of focal adhesion plaques, and stress fibers, spreading and wound closure in TgPED fibroblasts. Thus, PED/PEA-15 recruits activated ERK1/2 into the cytoplasm where it contributes to deregulate RhoA activity, localization, and function, thereby impairing cell motility (Fig. 10).

It has been recently described that ERK promotes Rho-dependent focal adhesion formation by phosphorylating and suppressing p190A Rho GAP (Pullikuth and Catling, 2010), suggesting that selective coupling of active ERK to distinct substrates and/or scaffolding protein could be responsible for alternative assembly or disassembly of cytoskeletal structures. Consistent with this hypothesis, the cellular abundance of the ERK interactor PED/PEA-15 may affect substrate availability and modulation of RhoA function.

Interestingly, fibroblasts isolated from KO mice displayed an increased spreading compared to control cells. These results strongly support a role of PED/PEA-15 in the regulation of cell motility. Moreover, the control exerted by PED/PEA-15 on cell migration is not restricted to fibroblasts, as it was already shown that PED/PEA-15 controls motility in astrocytes (Renault-Mihara et al., 2006).

Finally, the histological analysis of bioptic specimens from TgPED wounded skin showed a remarkable reduction in fibroblasts and in collagen fiber content, as well as a delay in re-epithelization of the wounded surface, which is consistent with a reduction of cellular motility. However, these alterations were accompanied by a reduced thickness of granulation tissue and a persistent infiltration of inflammatory cells. Conversely, skin repair was accelerated in KO mice, which also displayed an increased number of fibroblasts and collagen fibers at the wound site. Collectively, these data suggest that PED/PEA-15 is able to impair fibroblast functions, also in vivo. Since PED/PEA-15 may as well affect motility and/or function of other cell types (keratinocytes, endothelial cells, macrophages, etc.), we cannot exclude the possibility that the wound healing phenotype may be contributed by alterations different from those exclusively related to fibroblast motility. Whether other factors, including the response of inflammatory cells and/or angiogenesis contribute to impaired wound healing in TgPED mice is currently under investigation in our laboratory. Moreover, it should be pointed out that wound closure has been evaluated in TgPED mice, which, albeit glucose-intolerant and insulin-resistant, do not display overt diabetes (Vigliotta et al., 2004), raising the possibility that PED/PEA-15 effect is independent of the metabolic derangement occurring in diabetic conditions.

Thus, considering the different known cellular function of PED/PEA-15 in a chronic disorder such as diabetes (Condorelli et al., 2001; Vigliotta et al., 2004; Valentino et al., 2006), the observation that PED/PEA-15 regulates fibroblast motility may have a significant impact on future investigation of its role in diabetic complications.
