# Supplementary material for: PED/PEA-15 Controls Fibroblast Motility and Wound Closure by ERK1/2-Dependent Mechanisms
Source: J Cell Physiol. 2011 Jul 21;227(5):2106–16. doi: 10.1002/jcp.22944 (PMC3306794; doi:10.1002/jcp.22944)
Supplement: Supplementary file 4 [file jcp0227-2106-SD4.doc]

Supplementary videos legend

SupplementaryVideo 1 (WT). Confluent monolayers of fibroblasts from WT mice were subjected to scratch assays, as described in Materials and Methods. Fibroblast motility was then assessed by quantitative analysis of images acquired in TLM experiments (see Material and Methods) following the scratch and images were recorded with a time interval of 10 min for 24 h. Time stamp and scale bar are included in the video.

SupplementaryVideo 2 (TgPED). Confluent monolayers of fibroblasts from TgPED mice were subjected to scratch assays, as described in Materials and Methods. Fibroblast motility was then assessed by quantitative analysis of images acquired in TLM experiments (see Material and Methods) following the scratch and images were recorded with a time interval of 10 min for 24 h. Time stamp and scale bar are included in the video.

SupplementaryVideo 3 (KOPED). Confluent monolayers of fibroblasts from KO mice were subjected to scratch assays, as described in Materials and Methods. Fibroblast motility was then assessed by quantitative analysis of images acquired in TLM experiments (see Material and Methods) following the scratch and images were recorded with a time interval of 10 min for 24 h. Time stamp and scale bar are included in the video.
